# Correlation between thrombocytopenia and adverse outcomes in patients with atrial fibrillation: a systematic review and meta-analysis

**DOI:** 10.3389/fcvm.2024.1383470

**Published:** 2024-12-03

**Authors:** Qiuhua Ding, Wenlin Xu, Yaoyao Chen, Sijie Chang, Jinhua Zhang

**Affiliations:** Department of Pharmacy, Fujian Maternity and Child Health Hospital College of Clinical Medicine for Obstetrics & Gynecology and Pediatrics, Fujian Medical University, Fuzhou, China

**Keywords:** atrial fibrillation, thrombocytopenia, adverse outcomes, ischemic stroke/systemic embolism, major bleeding, all-cause mortality

## Abstract

**Background:**

Thrombocytopenia is often associated with adverse outcomes in patients with atrial fibrillation. Therefore, we conducted a meta-analysis to comprehensively assess the impact of thrombocytopenia on ischemic stroke/systemic embolism, major bleeding and all-cause mortality in patients with atrial fibrillation.

**Methods:**

Two electronic databases, PubMed and Web of Science, were systematically searched from their inception to December 1, 2023, including the studies on the correlation between atrial fibrillation patients with thrombocytopenia and adverse outcomes. Relevant data was extracted, literature quality was evaluated, meta-analysis was performed by using REVMAN 5.4 software, and the results were reported with odds ratio (OR) of 95% confidence interval (CI).

**Results:**

A total of 12 studies included 73,824 patients with atrial fibrillation (average age: 72.67, males: 42,275, 57.3%), among them, there were 7,673 patients combined with thrombocytopenia. The average follow-up time of these studies was 87 days to 55 months. Compared to no thrombocytopenia, atrial fibrillation patients combined with thrombocytopenia have a significant risk reduction of ischemic stroke/systemic embolism [OR: 0.79, 95% CI: (0.69, 0.91); *P* < 0.01]. Nevertheless, the risk of both major bleeding [OR: 1.51, 95% CI: (1.20, 1.79), *P* < 0.01] and all-cause mortality [OR: 1.40, 95% CI: (1.23, 1.61); *P* < 0.01] is significantly higher in thrombocytopenia group.

**Conclusions:**

Thrombocytopenia has an important impact on the prognosis of patients with atrial fibrillation. Thrombocytopenia is significantly associated with a lower risk of ischemic stroke/systemic embolism but a higher risk of major bleeding and all-cause mortality. Attention to thrombocytopenia and optimization of treatment may be the effective way to improve the prognosis of atrial fibrillation with thrombocytopenia.

**Systematic Review Registration:**

https://www.crd.york.ac.uk/, PROSPERO Registration Number: (CRD42023459916).

## 1 Background

Atrial fibrillation (AF) is the most common persistent arrhythmia, with serious complications such as thromboembolism, stroke and heart failure. AF also significantly increases risks of myocardial infarction, cognitive dysfunction, dementia and death, which seriously affects AF patients’ quality of life (1–3). The treatment of AF, besides controlling rhythm and ventricular rate, the most important thing is to prevent stroke. The use of anticoagulants is one of the main management measures for AF (4, 5). Although anticoagulant therapy can reduce the occurrence of thromboembolic events, it is often accompanied by a certain risk of bleeding. Particularly for some patients with high bleeding risk such as AF with thrombocytopenia, the use of anticoagulants lacks safety data, and this therapeutic measure will face great challenges.

CHA₂DS₂-VASC scoring tool and HAS-BLED prediction model have been widely used for stratifying risk assessment of thromboembolism or hemorrhage in atrial fibrillation (2, 5, 6). Nevertheless, other clinical features which are not included in these scores may also be the risk factors for thromboembolism or hemorrhage in patients with AF or Nonvalvular Atrial Fibrillation (NVAF), such as abnormal platelet count (7), low body weight or body mass index (BMI) (8), low creatinine clearance (CrCl) value (9) etc. Of particular noteworthy, various studies have shown that abnormal platelet levels may affect the clinical outcome of AF patients (10–12). Platelets are produced by the bone marrow, and platelet count (PLT) is one of the main markers of platelet activity, which are often used to judge the bleeding condition or thrombotic disease in clinic. There are no clear data on the prevalence of AF with thrombocytopenia, and the 2023 ACC/AHA/ACCP/HRS guideline for the diagnosis and management of atrial fibtrllation do not provide any treatment recommendations for AF patients with thrombocytopenia (13). Moreover, these subjects were excluded from large randomized trials of oral anticoagulants, and current studies on the correlation between platelet levels and adverse AF outcomes also showed conflicting conclusions (14, 15). Therefore, there are many uncertainties and clinical risks with the treatment and management of this particular group of AF patients.

At present, the study on the correlation between patients with AF and thrombocytopenia has been insufficient, and no systematic review and meta-analysis has summarized the possible adverse outcomes of ischemic stroke/systemic embolism, major bleeding and all-cause mortality in this particular population. Therefore, we conducted a systematic review and meta-analysis of current relevant studies on patients with AF and thrombocytopenia to comprehensively evaluate the impact of thrombocytopenia on adverse outcomes in AF, so as to provide guidance for clinical treatment of AF complicated with thrombocytopenia.

## 2 Method

This systematic review and meta-analysis was conducted under the Preferred Reporting Project (PRISMA) (16), registration number: CRD 42023459916.

### 2.1 Search strategy

**Figure 1:**
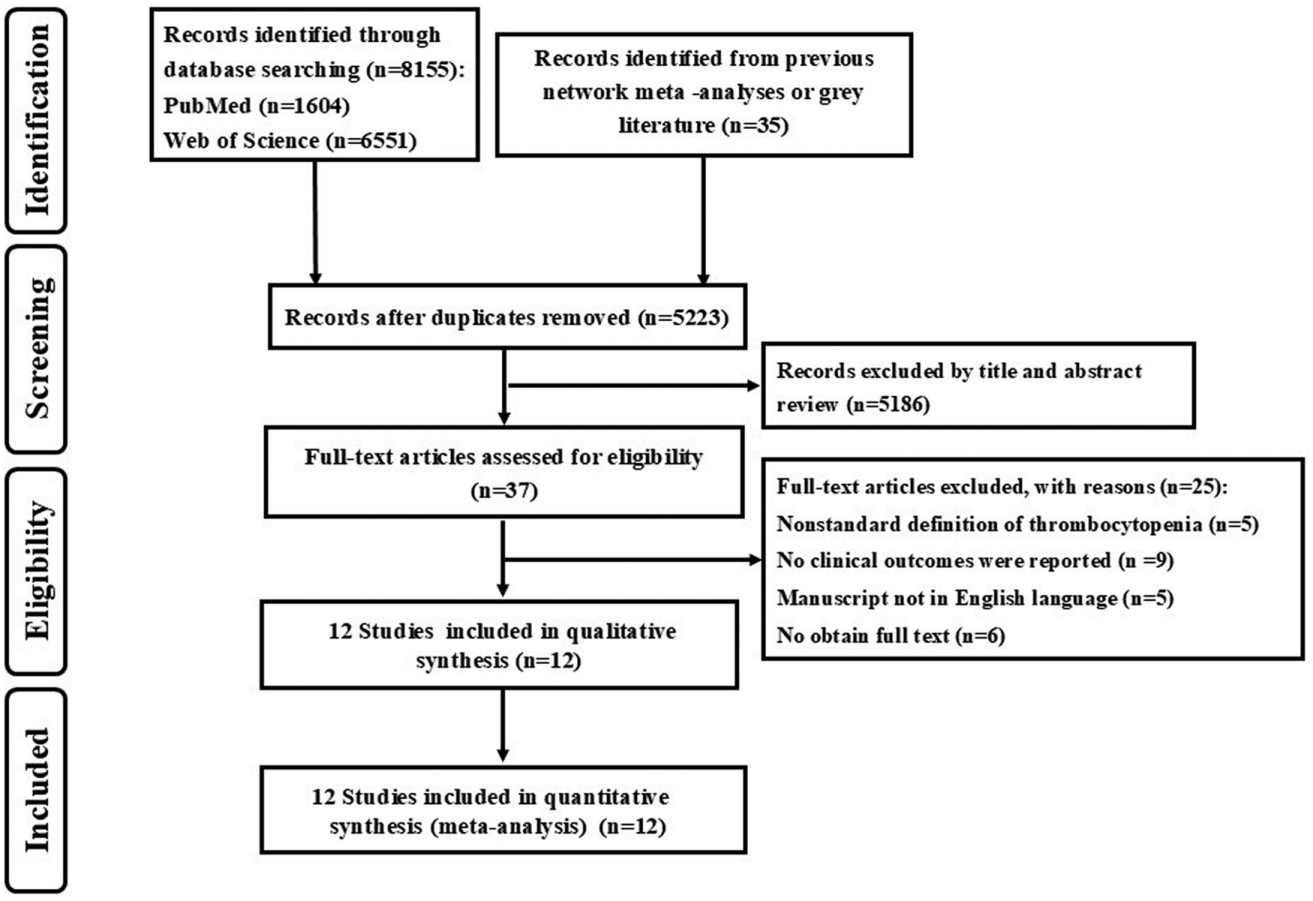
Flow chart of the search strategy.

Searching PubMed and Web of Science databases for conducting literature search till December 1, 2023 (see the flow chart in Figure 1 for the complete searching strategy), with no restrictions on publication status. For a comprehensive literature search, we also manually search for the references included in studies, review articles, and editorials/letters.

### 2.2 Inclusion and exclusion criteria

Inclusion criteria: (1). Retrospective or prospective cohort study; (2). Patients with AF and concurrent thrombocytopenia; (3). The study reported the platelet level of the patient, which was in line with the diagnostic criteria for thrombocytopenia provided by the international consensus report (17): Thrombocytopenia is usually defined as platelet count less than 100 × 10^9^/L in Asian; In Europe and America, thrombocytopenia is usually defined as platelet count less than 150 × 10^9^/L. (4). Studies have reported the correlation between thrombocytopenia as well as one or more of the following outcomes: ischemic stroke/systemic embolism, bleeding events or mortality in AF.

Exclusion criteria: (1). Full text cannot be obtained; (2). Non-English literature; (3). The definition of thrombocytopenia is not international standardized; (4). No clinical outcomes were reported in AF.

### 2.3 Data extraction

Data and literature were collected and extracted independently by two reviewers (QD and WX), and if there was any dispute, it would be discussed and resolved by a third researcher to reach a consensus. From each study, we extracted the study type, treatment, study population, average age, male proportion, country, follow-up period and outcome indicator (ischemic stroke/systemic embolism, bleeding events, mortality etc.).

### 2.4 Quality evaluation

The Newcastle-Ottawa scale was used to measure three aspects of a study: selection of subjects, comparability between groups, and outcome measurement. Except for comparability, which can score up to 2 points, the rest are 1 point, the highest score is 9 points, 7 to 9 means high quality, 4 to 6 means medium quality, and the score below 4 means low quality.

### 2.5 Clinical outcomes

#### 2.5.1 Primary outcome

①Schemic stroke/systemic embolism: schemic stroke or systemic embolism.

Schemic stroke (18): Schemic stroke are caused by narrowing or clogging of arteries leading to the brain, or by a blood clot or piece of debris breaking away from a blood vessel and causing a blockage in one of the brain arteries, is the most common type of stroke.

Systemic embolism (19): Systemic embolism was defined as signs and symptoms of peripheral ischemia associated with a positive imaging test result.

②Major bleeding (20): Bleeding leading to a ≥ 2 g/dl fall in hemoglobin or a transfusion of ≥2 units of packed red blood cells or whole blood; Bleeding into a critical site (intracranial, intraspinal, intraocular, retropertoneal, intra-anicwlar, pericardia, or intramuscular with compartment syndrome), or Bleeding leading to death.

③All-cause mortality: All-cause mortality is defined as death from any cause (21).

#### 2.5.2 Secondary outcome

①Combined bleeding events: combined major, minor bleeding and clinically relevant non-major bleeding with other types of bleeding to evaluate the predictors of significant bleedings.

②Minor bleeding (22): any other overt bleeding episodes not meeting the criteria for major bleeding or non-major clinically relevant bleeding events.

③Gastrointestinal hemorrhage (23): bleeding of the digestive tract from the esophagus to the anus.

④Intracranial hemorrhage (24): bleeding within the skull. Subtypes are intracerebral bleeds (intraventricular bleeds and intraparenchymal bleeds), subarachnoid bleeds, epidural bleeds, and subdural bleeds.

⑤Clinically relevant non-major bleeding (25): any sign of hemorrhage that did not fulfill major bleeding criteria but met at least one of the following: required medical intervention, led to hospitalization, or prompted face to face evaluation.

### 2.6 Statistical analysis

The analysis was performed by using REVMAN 5.4 and Stata 16.0 software, and CI was used as the combined effect size of the binary classification. Odds ratios (OR) were calculated for all outcomes using the pooled effect of each therapy according to the Mantel–Haenszel method for random effects. [*I*^2^] was used to evaluate the heterogeneity among the trials (26), *I*^2^ ≤ 50% indicated that there was no statistical heterogeneity among the studies, *I*^2^ > 50% suggested that there was heterogeneity among studies. The publication bias of the literature included for outcome measures was assessed by Egger's test (27). *P* < 0.05 was considered statistically significant.

## 3 Results

### 3.1 Literature retrieval

The searching strategy had identified 5,223 studies (Figure 1). Of these, 37 potentially relevant studies were identified for full text assessment. After excluding 25 studies, all the rest 12 studies met the inclusion criteria. In the report OR research, 10 studies examined the association between thrombocytopenia and ischemic stroke/systemic embolism, 9 studies examined the association between thrombocytopenia and major bleeding, and 7 studies examined the association between thrombocytopenia and all-cause mortality.

### 3.2 Basic characteristics and quality evaluation of included studies

The 12 studies included in this systematic review has been analyzed, including 8 studies on Asian populations, 4 studies on European and American populations. A total of 73,824 patients with atrial fibrillation and thrombocytopenia were analyzed, with average age of 72.67, 57.3% male, and the average follow-up ranging from 87 days to 55 months. The baseline characteristics of 12 studies included was shown in Table 1, The quality evaluation of 12 studies included was used by the Newcastle-Ottawa scale as Supplementary Table S1.

**Table 1:** Baseline characteristics of each study included.

Authors	Study period	Country	Study design	AF type	Administration	Number of patients (n)	Age (mean, years)	Sex (male,%)	Heart failure	Median follow-up (months)
						Thrombocytopenia/Normal Platelet Count				
Agnieszka Janion-Sadowska et al.	January 2012–July 2015	Poland	Prospective cohort	AF	NOACs	62/62	70.5/70.0	20 (32.3)/21 (33.9)	26 (41.9)/26 (41.9)	55
Chun-Li Wang et al.	2010–2017	Taiwan	Retrospective cohort	AF	OACs	367/7872	78.6/76.9	209 (56.9)/4077 (51.8)	202 (55.0)/3159 (40.1)	46
Daniele Pastori et al.	2016–2018	Italy	Multicenter prospective cohort study	AF	OACs	592/4623	75.6/74.9	431 (72.8)/2416 (52.3)	102 (17.2)/706 (15.3)	19.2
Eitaro Kodani et al.	2017–2019	Japan	Prospective observational	NVAF	VKA or Antiplatelet	130/313	74.7/68.9	95 (73.1)/191 (61.0)	69 (53.1)/103 (32.9)	24
Jiesuck Park et al.	2000–2013	Korea	Prospective cohort	NVAF	OACs	2,656/8322	73.5/73.3	1,803 (67.9)/5174 (62.2)	424 (15.9)/1074 (12.9)	42.6
Tuomas Kiviniemi et al.	2011–2012	Finland	Prospective cohort	AF	VKA or Antiplatelet	99/762	74/73	84 (85)/518 (68)	21 (21)/16 (21)	12
Varun Iyengar, et al.	2015–2020	USA	Retrospective cohort	AF	OACs	274/796	72/72	170 (62.0)/491 (61.7)	99 (36.1)/67 (8.4)	12
Wenlin Xu et al	2016–2020	China	retrospective cohort	AF	NOACs	154/3804	68.7/62.9	82 (53.0)/2286 (60.1)	19 (12.4)/141 (3.7)	-
Xiaochun Zhang et al.	January 2016–December 2018	China	Prospective observational cohort	AF	DAPT or NOACs undergoing LAAO	32/160	69.4/69.2	8 (25.0)/43 (26.8)	2 (3.13)/5 (3.13)	24
Yoav Michowitz et al.	June 2006–January 2018	Israel	Monocentric observational retrospective	NVAF	OACs	1,617/9739	78.7/76.6	1,163 (71.9)/4893 (50.2)	579 (35.8)/2846 (29.2)	40.6
Yung-Hsin Yeh et al.	July 2001–September 2018	Taiwan	Retrospective cohort	AF	OACs	1,665/29147	74.5/70.6	841 (50.5)/17024 (58.4)	92 (5.54)/887 (3.04)	-
Yurong Xiong et al.	February 2015–December 2017	China	Multicenter, prospective and observational	NVAF	Dabigatran after radiofrequency ablation	25/551	63.8/62.8	216 (66.9)/216 (66.9)	5 (20.0)/36 (11.1)	3

NOACs, new oral anticoagulants; VKA, warfarin; OACs, oral anticoagulants, including warfarin; NOACs, antiplatelet, aspirin, clopidogrel; DAPT, dual platelet therapy; LAAO, left atrial appendage occlusion.

### 3.3 Association between thrombocytopenia and ischemic stroke/systemic embolism

**Figure 2:**
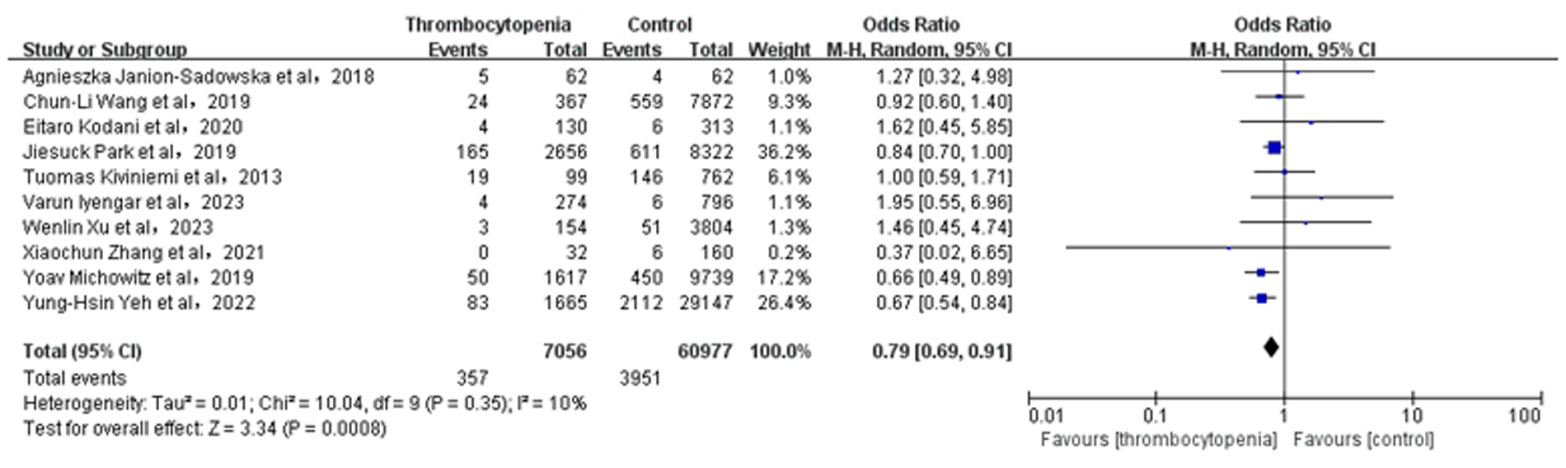
Association of thrombocytopenia and ischemia stroke/systemic embolism.

A total of 10 studies with 63,005 participants involved examined the association between thrombocytopenia and ischemic stroke/systemic embolism (Figure 2). Thrombocytopenia was significantly associated with a lower risk of ischemic stroke/systemic embolism in patients with AF [OR: 0.79, 95% CI: (0.69, 0.91), *P* = 0.0008], with low heterogeneity ([*I*^2^] = 10%). The Egger's test revealed no evidence of publication bias (Supplementary Table S2, *P* = 0.0891). Given that the impact of different anticoagulant regimens on outcomes, we performed a subgroup analysis of ischemic stroke/systemic embolism according to the anticoagulant regimen [OR: 0.79, 95% CI: (0.68 −0.91), *P* = 0.002] (Supplementary Figure S1), which are concurred with the overall analysis.

### 3.4 Association between thrombocytopenia and major bleeding

**Figure 3:**
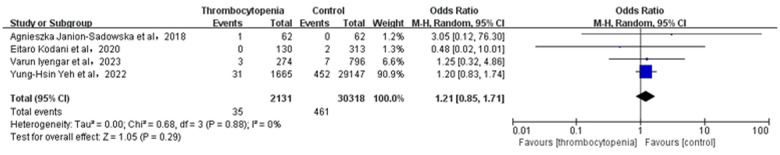
Association of thrombocytopenia and major bleeding.

A total of 9 studies with 57,055 participants involved examined the association between thrombocytopenia and major bleeding (Figure 3). Thrombocytopenia was significantly associated with a higher risk of major bleeding [OR: 1.51, 95% CI: (1.20, 1.79), *P* = 0.0002], with not high heterogeneity ([*I*^2^] = 41%). Given the mild heterogeneity, we performed a sensitivity analysis to explore a cause of heterogeneity by excluding the study [Varun Iyengar et al. (10)], and found that thrombocytopenia compared with control was associated with a significant increased risk of major bleeding [OR: 1.34; 95% CI: (1.18, 1.52), *P* < 0.00001] (Supplementary Figure S4), with not heterogeneity [(*I*^2^) = 0%]. Meanwhile, we performed a subgroup analysis of major bleeding according to the anticoagulant regimen [OR: 1.47, 95% CI: (1.20 −1.79), *P* = 0.0002] (Supplementary Figure S2), which is concurred with the overall analysis. The Egger's test revealed no evidence of publication bias (Supplementary Table S2, *P* = 0.8912).

### 3.5 Association between thrombocytopenia and combined bleeding events

**Figure 4:**
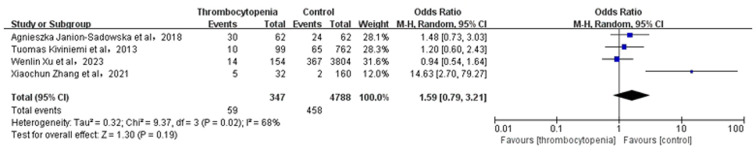
Association of thrombocytopenia and bleeding events.

A total of 6 studies with 28,799 participants involved examined the association between thrombocytopenia and combined bleeding events (Figure 4). Thrombocytopenia was significantly associated with a higher risk of combined bleeding events in patients with AF [OR: 1.51, 95% CI: (1.27, 1.81), *P* < 0.00001], with not high heterogeneity [(*I*^2^) = 43%]. The Egger's test showed publication bias (Supplementary Table S2, *P* = 0.0049), therefore, the result may be not very reliable in combined bleeding events.

### 3.6 Association between thrombocytopenia and minor bleeding

**Figure 5:**
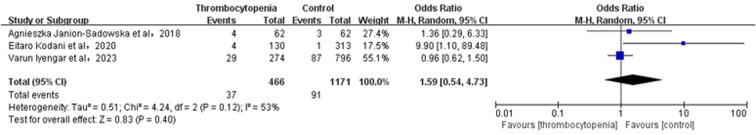
Association of thrombocytopenia and minor bleeding.

A total of 4 studies with 5,135 participants involved examined the association between thrombocytopenia and minor bleeding (Figure 5). Overall, the risk of minor bleeding in AF combined with thrombocytopenia was similar to that in AF [OR: 1.59, 95% CI: (0.79, 3.21), *P* = 0.19], with significant heterogeneity [(*I*^2^) = 68%]. Due to high heterogeneity, we performed the sensitivity analysis of minor bleeding, and found that thrombocytopenia was associated with a significant increased risk of minor bleeding [OR: 1.14; 95% CI: (0.79, 1.66), *P* = 0.49] compared with control group, with significant heterogeneity [(*I*^2^) = 0%] by excluding the study (28) (Supplementary Figure S5). The Egger's test revealed publication bias (Supplementary Table S2, *P* = 0.0024), therefore, the result is not very reliable in minor bleeding.

### 3.7 Association between thrombocytopenia and gastrointestinal hemorrhage

**Figure 6:**
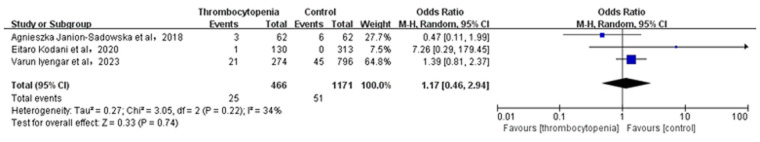
Association of thrombocytopenia and gastrointestinal hemorrhage.

A total of 3 studies with 1,637 participants involved examined the association between thrombocytopenia and gastrointestinal hemorrhage (Figure 6). Overall, the risk of gastrointestinal hemorrhage in AF combined with thrombocytopenia was similar to that in AF [OR: 1.26, 95% CI: (0.46, 2.94), *P* = 0.74], with low heterogeneity [(*I*^2^) = 34%].The Egger's test showed no evidence of publication bias (Supplementary Table S2, *P* = 0.5394).

### 3.8 Association between thrombocytopenia and intracranial hemorrhage

**Figure 7:**
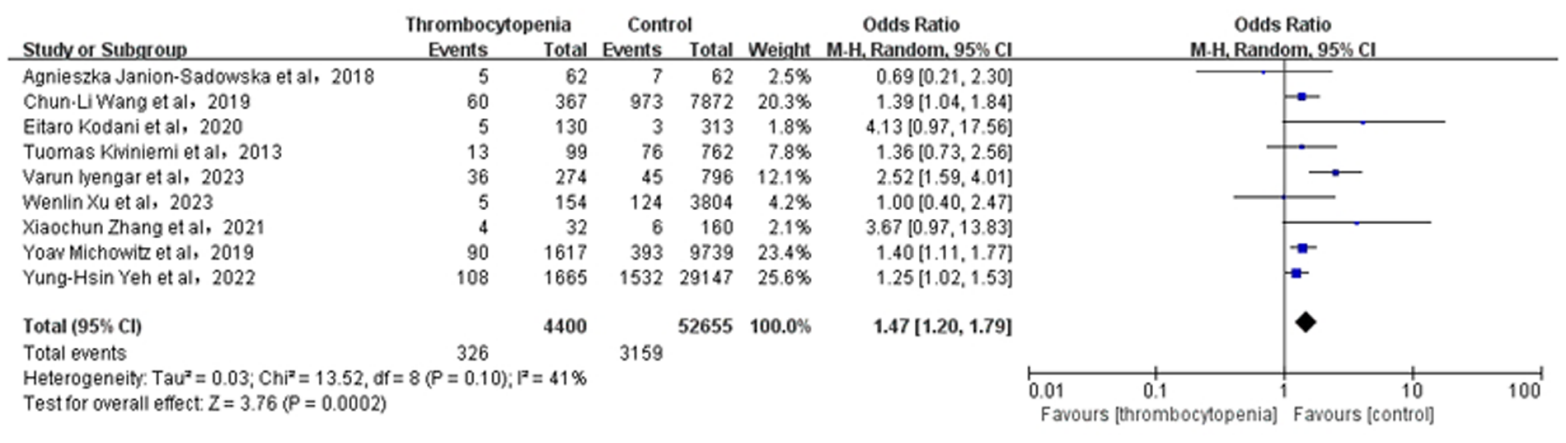
Association of thrombocytopenia and intracranial hemorrhage.

A total of 4 studies with 32,449 participants involved examined the association between thrombocytopenia and intracranial hemorrhage (Figure 7). Overall, the risk of intracranial hemorrhage in AF combined with thrombocytopenia was similar to that in AF [OR: 1.21, 95% CI: (0.85, 1.71), *P* = 0.29], with no heterogeneity [(*I*^2^) = 0%]. The Egger's test showed no evidence of publication bias (Supplementary Table S2, *P* = 0.9933).

### 3.9 Association between thrombocytopenia and clinically relevant non-major bleeding

**Figure 8:**
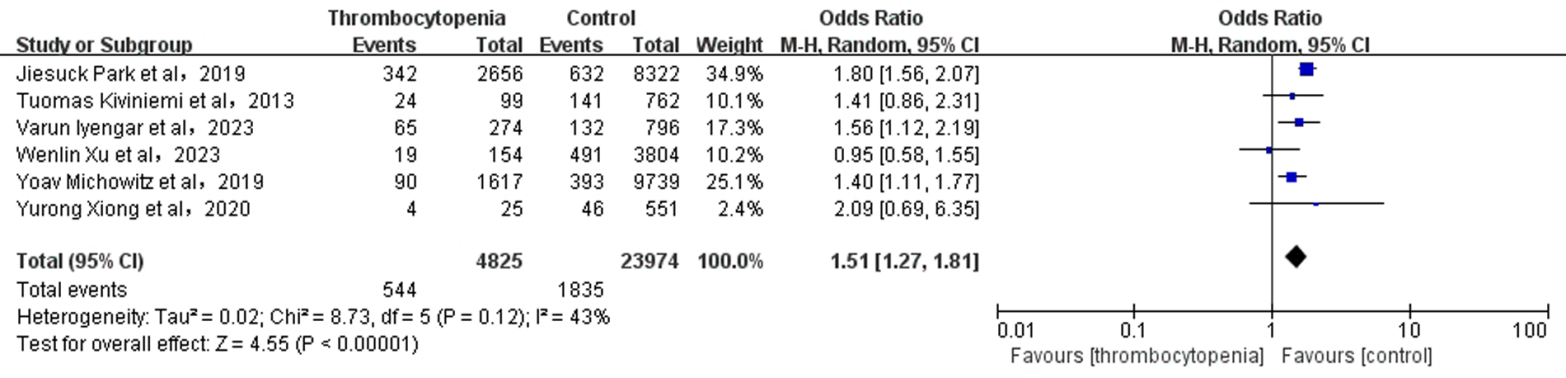
Association of thrombocytopenia and clinically relevant non-major bleeding.

A total of 3 studies with 1637 participants involved examined the association between thrombocytopenia and clinically relevant non-major bleeding (Figure 8). Overall, the clinically relevant risk of non-major bleeding in AF combined with thrombocytopenia was similar to that in AF [OR: 1.59, 95% CI: (0.54, 4.73), *P* = 0.40]. Heterogeneity was relatively high [(*I*^2^) = 53%]. Due to the high heterogeneity, we also conducted a sensitivity analysis by excluding the study [Eitaro Kodani et al. (29)] and found that thrombocytopenia was associated with a nonsignificant increased risk of clinically relevant non-major bleeding [OR: 0.99; 95% CI: (0.65, 1.52)] compared with control group (Supplementary Figure S6). The Egger's test displayed no evidence of publication bias (Supplementary Table S2, *P* = 0.0997).

### 3.10 Association between thrombocytopenia and all-cause mortality

**Figure 9:**
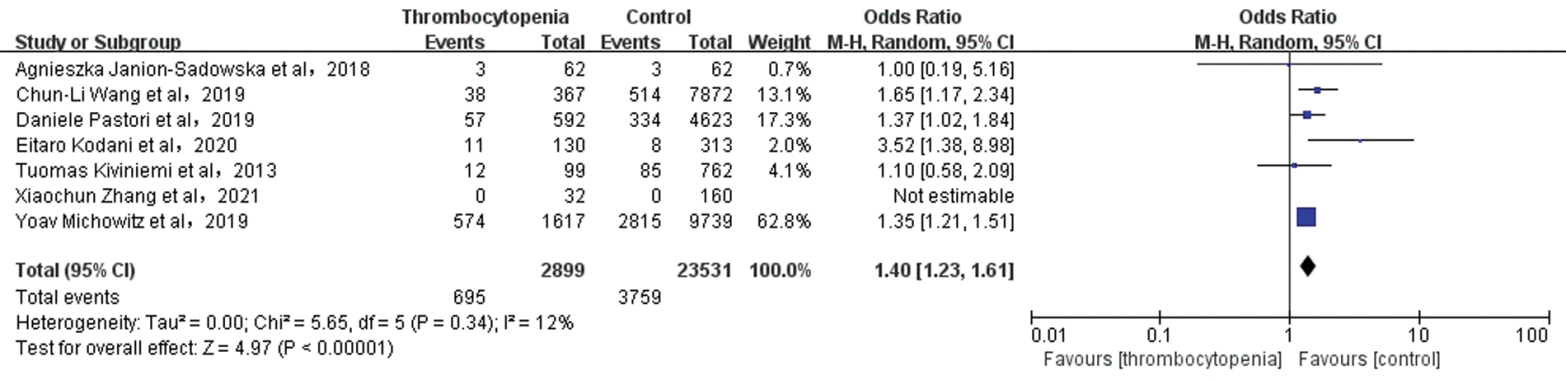
Association of thrombocytopenia and all-cause mortality.

A total of 7 studies with 26,430 participants involved examined the association between thrombocytopenia and all-cause mortality (Figure 9). Thrombocytopenia was significantly associated with a higher risk of all-cause mortality [OR: 1.40, 95% CI: (1.23, 1.61), *P* < 0.00001], with no significant heterogeneity [(*I*^2^) = 12%]. The Egger's test showed no publication bias (Supplementary Table S2, *P* = 0.2872). To better examine the relationship between thrombocytopenia and all-cause mortality, we also performed a subgroup analysis according to the anticoagulant regimen. We found that thrombocytopenia was significantly associated with a higher risk of all-cause mortality [OR: 1.40, 95% CI: (1.23 −1.61), *P* < 0.00001] (Supplementary Figure S3), which is concurred with the overall analysis.

## 4 Discussion

At present, there are no abundant randomized studies on the relationship between the prognosis and PLT level in AF patients. Meanwhile, the studies on thrombocytopenia as a prognostic indicator in patients with AF are limited to a handful of observational studies or case reports, which also show conflicting results. Kodani (29) reported that platelet levels are not associated with any adverse outcomes in AF, whereas Park found that thrombocytopenia was associated with a lower risk of stroke but a higher risk of bleeding (30). Due to insufficient studies on the association between AF and thrombocytopenia, furthermore, no systematic review and meta-analysis has summarized the adverse outcomes of ischemic stroke/systemic embolism, bleeding events, and mortality that may occur in this particular AF population. Therefore, for the first time, we conducted a systematic review and meta-analysis of current studies on the association between AF outcomes and thrombocytopenia. In order to comprehensively elaborate the correlation between thrombocytopenia and prognosis in AF, and provide guidance for safer and more effective clinical management of AF complicated with thrombocytopenia.

So far, there are no clear epidemiological survey data to reveal the prevalence of AF with thrombocytopenia. This meta-analysis showed that patients with thrombocytopenia account for 10.39% of the population with AF, which is consistent with the prevalence of thrombocytopenia reported in previous study ranging from 6% to 24% (31). In the present meta-analysis of 73,824 patients with AF, our main findings are as follows: (1). Thrombocytopenia is significantly associated with a higher risk of combined bleeding events and mortality; (2). Thrombocytopenia is significantly associated with a lower risk of ischemic stroke/systemic embolism; (3). Although it's not statistically significant, the thrombocytopenia might potentially increase the risk of minor bleeding, gastrointestinal hemorrhage, intracranial hemorrhage, and other clinically relevant non-major bleeding. In a word, the present study revealed that thrombocytopenia was closely associated with adverse clinical outcomes in patients with AF. Therefore, screening the comorbidities of AF patients and optimizing the treatment regimen of AF with thrombocytopenia may be an effective measure to reduce adverse outcomes in AF.

### 4.1 Thrombocytopenia and ischemic stroke/systemic embolism

Platelets play a crucial role in the complex clotting process. They release clotting factors and activate the clotting pathway by aggregating in injured blood vessels, and eventually form thrombus (32). However, excessive or rapid formation of thrombus may cause vascular obstruction, affect blood circulation, even lead to ischemic stroke/systemic embolism, myocardial infarction and other serious thrombotic diseases (33). On the contrary, low platelet count or decreased coagulation function will increase the risk of bleeding (34). A study on the correlation between PLT counts and stroke occurrence and prognosis found that PLT counts were positively correlated with the risk of ischemic stroke, elevated platelet count (>300 × 10⁹/L) was a risk factor for ischemic stroke, and low PLT count (<100 × 10⁹/L) was associated with a reduced risk of ischemic stroke (35). Another study has found out the similar results, low platelet count (<50 × 10⁹/L) could reduce the risk of venous thromboembolism (36). This meta-analysis also found that AF patients with thrombocytopenia had a significantly reduced risk of ischemic stroke/systemic embolism compared to general AF, which is consistent with the conclusions of the above studies.

However, other studies observed that thrombocytopenia could not reduce thromboembolic events, especially in the case of AF complications (37, 38). Thrombocytopenia might be a causal factor in an ischemic stroke, a risk factor for hemorrhagic stroke (39). Due to limited data on AF patients with thrombocytopenia, whether low PLT can protect AF patients against ischemic stroke/systemic embolic events remains to be further studied.

### 4.2 Thrombocytopenia and major bleeding

The central role of platelets in the clotting process is self-evident. The decrease in platelet number or function will increase the clotting time, and the main clinical consequence is bleeding caused by impaired primary hemostatic function and platelet embolism formation (40). An observational study has confirmed that a lower platelet count was related with an increased risk of spontaneous bleeding (41). The key determinants of the anticoagulant bleeding rate in AF are age, previous thrombosis and bleeding events (42). Then, could platelet level be another determinant of bleeding events in AF? A previous research have verified that platelet level was an independent predictor of bleeding events (43). Moreover, the degree of thrombocytopenia was positively correlated with the risk of bleeding, more severe thrombocytopenia (<75 × 10⁹/L) was associated with an increased risk of major bleeding (10). Our meta-analysis similarly displayed that AF patients with thrombocytopenia had a significantly increased risk of bleeding events compared to AF patients without thrombocytopenia, which is consistent with the conclusions of previous cohort studies (44, 45). We also found that AF patients with thrombocytopenia had a greater tendency to minor bleeding, gastrointestinal hemorrhage, intracranial hemorrhage, and clinically relevant non-major bleeding, although the correlation was not statistically significant, which might be due to the small number of studies we included, limited by the insufficient population. Future studies need to enroll more literature and conduct subgroup analysis according to the severity of thrombocytopenia, so as to obtain more accurate data for stratified prediction of bleeding risk in AF patients combined with thrombocytopenia.

### 4.3 Thrombocytopenia and all-cause mortality

Thrombocytopenia can be divided into primary and acquired thrombocytopenia. Primary thrombocytopenia is mostly caused by chronic blood diseases. The causes of acquired thrombocytopenia may include trauma, surgery, infection, drugs, radiotherapy, immune dysfunction, and nutritional disorders (46). Multiple studies have shown a significant association between thrombocytopenia and increased risk of all-cause mortality (47–51). Particularly, another study showed that chronic kidney disease, active cancer, and liver cirrhosis, bone marrow disorders which are frequent features of patients with AF were significantly associated with thrombocytopenia (52).

The risk of mortality in patients with AF is 1.5 to 1.9 times the mortality of the normal population (53), and the types of mortality can be divided into cardiovascular death and non-cardiovascular death. The risk factors mainly include infection, cancer, blood uric acid, diabetes, hypertension, myocardial infarction and heart failure (54, 55). Although no studies investigated the specific reasons of mortality in AF with thrombocytopenia, the correlation between thrombocytopenia and mortality in AF may be mediated by thrombocytopenia affecting these major risk factors. The present meta-analysis found that AF patients with thrombocytopenia had a significant higher risk of mortality than AF patients without thrombocytopenia, which was consistent with previous studies. In addition, according to the research (56), the bleeding of AF patients was closely related to subsequent major unscrupulous cerebrovascular events (MACCE) and death, and preventing major bleeding could significantly avoid cardiovascular events and death. Our analysis has also shown similar results that AF complicated with thrombocytopenia had a significantly increased risk of bleeding events, major bleeding, and potentially increased risk of fatal bleeding such as intracranial hemorrhage, which might be one of the reasons of significantly increased risk of mortality in AF complicated with thrombocytopenia.

Therefore, thrombocytopenia increases the all-cause mortality. If AF is accompanied by thrombocytopenia, there is significant increase the additional mortality. Traditionally, the focus of improving the prognosis of AF is on preventing stroke, and anticoagulation therapy has been shown to significantly reduce thromboembolism and stroke-related mortality in AF (57). Our analysis suggests that screening and attention to comorbidities, such as thrombocytopenia, may be one of the effective ways to reduce the risk of mortality in patients combined with AF. What's more, the complex association between atrial fibrillation with thrombocytopenia and mortality needs further investigation.

## 5 Limitation

This systematic review and meta-analysis have some certain limitations: First, the number of studies and patients included were not enough, resulting in a lack of confidence in some outcome indicators, such as minor bleeding, gastrointestinal hemorrhage, intracranial hemorrhage and clinically relevant non-major bleeding, whose results were not statistically significant. Second, the baseline characteristics of patients are not uniform, study populations are from Asia, Europe or America. Therefore, the bias of unmeasured confounders may be present. Third, no high-quality RCT studies were available for inclusion in our analysis, further researches on this population are necessary to confirm the correlation between thrombocytopenia and prognosis in patients with AF.

## 6 Conclusion

Thrombocytopenia has an important impact on the prognosis of patients with atrial fibrillation. Thrombocytopenia is significantly associated with a lower risk of ischemic stroke/systemic embolism but a higher risk of major bleeding and all-cause mortality. Attention to thrombocytopenia and optimization of treatment may be an effective way to improve the prognosis of atrial fibrillation with thrombocytopenia.

## Data Availability

The original contributions presented in the study are included in the article/Supplementary Material, further inquiries can be directed to the corresponding author.

## Supplementary material

Supplementary Figure S1 Subgroup analysis of Ischemia stroke/systemic embolism.

Supplementary Figure S2 Subgroup analysis of Major bleeding.

Supplementary Figure S3 Subgroup analysis of All-cause mortality.

Supplementary Figure S4 Sensitivity analysis of Major bleeding.

Supplementary Figure S5 Sensitivity analysis of Minor bleeding.

Supplementary Figure S6 Sensitivity analysis of clinically relevant non-major bleeding.
