# Supplementary figures and images for: Correlation between thrombocytopenia and adverse outcomes in patients with atrial fibrillation: a systematic review and meta-analysis
Source: Front Cardiovasc Med. 2024 Dec 3;11:1383470. doi: 10.3389/fcvm.2024.1383470 (PMC11649656; doi:10.3389/fcvm.2024.1383470)

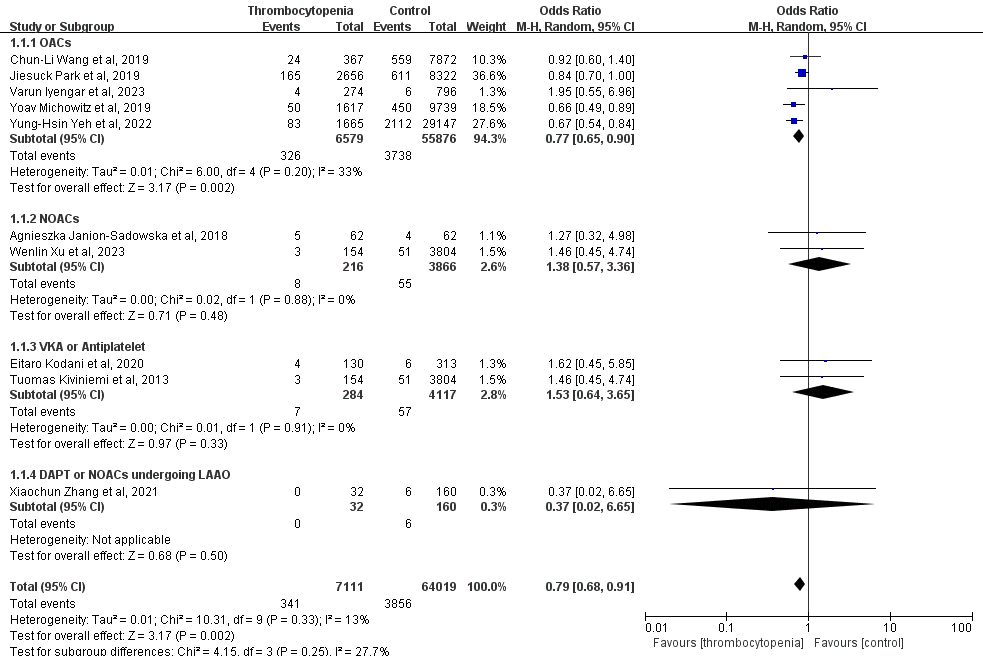

Supplement: Supplementary Figure S1 — Subgroup analysis of Ischemia stroke/systemic embolism. [file Image1.png]

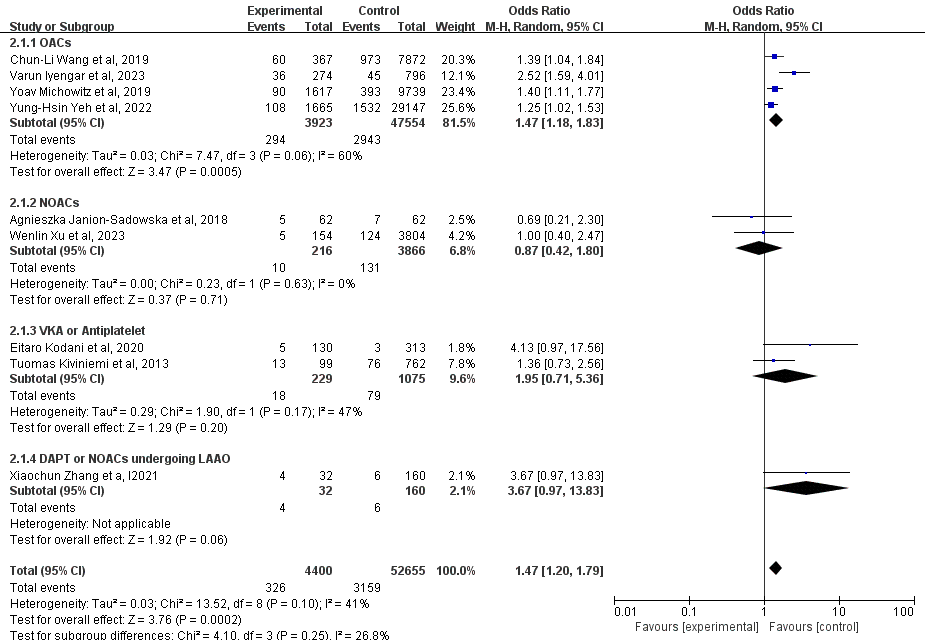

Supplement: Supplementary Figure S2 — Subgroup analysis of Major bleeding. [file Image2.png]

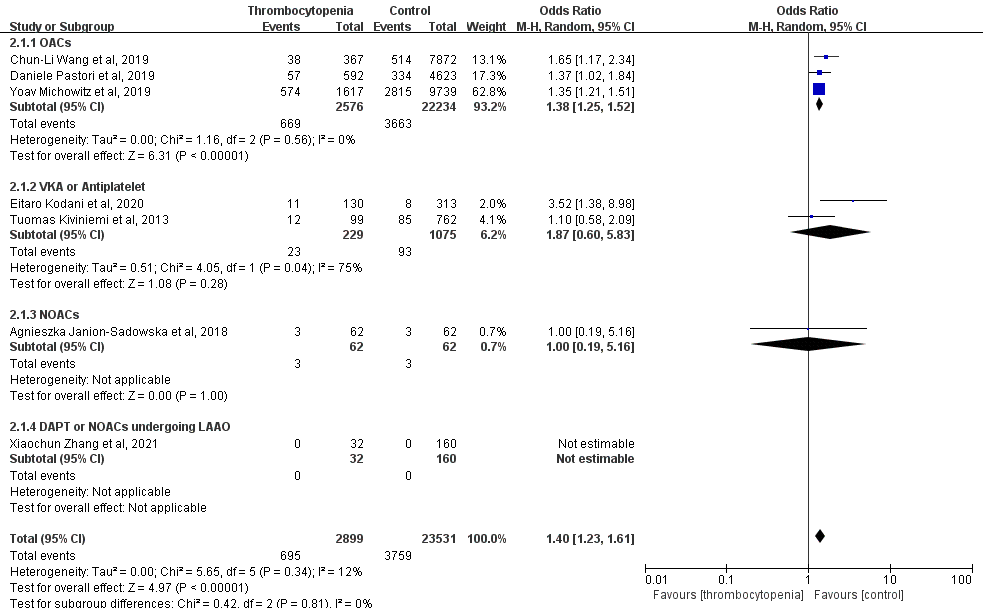

Supplement: Supplementary Figure S3 — Subgroup analysis of All-cause mortality. [file Image3.png]

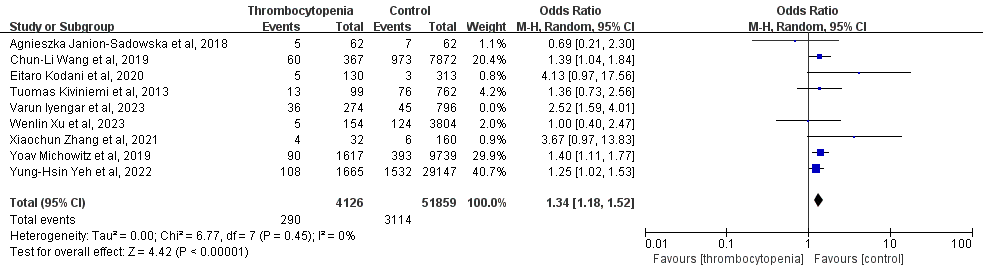

Supplement: Supplementary Figure S4 — Sensitivity analysis of Major bleeding. [file Image4.png]

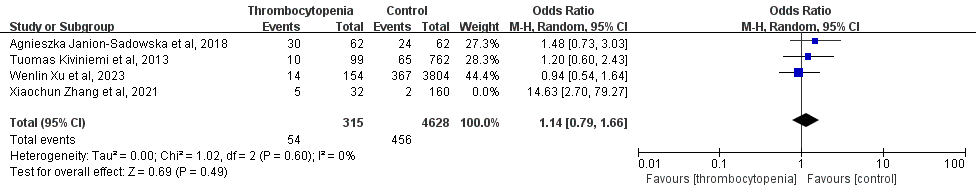

Supplement: Supplementary Figure S5 — Sensitivity analysis of Minor bleeding. [file Image5.png]

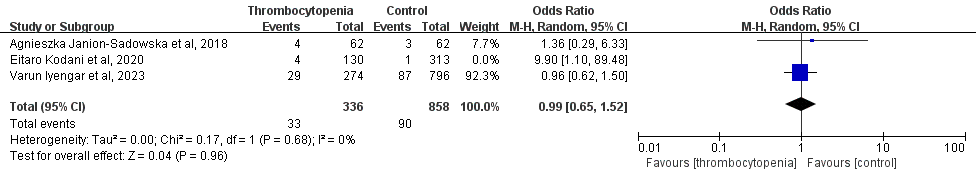

Supplement: Supplementary Figure S6 — Sensitivity analysis of clinically relevant non-major bleeding. [file Image6.png]
