# Supplementary material for: Correlation between thrombocytopenia and adverse outcomes in patients with atrial fibrillation: a systematic review and meta-analysis
Source: Front Cardiovasc Med. 2024 Dec 3;11:1383470. doi: 10.3389/fcvm.2024.1383470 (PMC11649656; doi:10.3389/fcvm.2024.1383470)
Supplement: Supplementary file 7 [file Table1.docx]

| **Supplementary Table S1. Quality evaluation of the eligible studies with Newcastle–Ottawa scale** | | | | | | | | | | |  |
| --- | --- | --- | --- | --- | --- | --- | --- | --- | --- | --- | --- |
|  |  |  |  |  |  |  |  |  |  |  |  |
| **Study** | **Research object selection** | | | | **Comparability between groups** | | **outcome measurement** | | | **Quality score** |  |
|  | **Representative-ness** | **Selection of non-exposed** | **Ascertainment of exposure** | **Outcome not present at start** | **Comparability on most important factors** | **Comparability on other risk factors** | **Assessment of outcome** | **Long enough follow-up (median≥1 year)** | **Adequacy (completeness) of follow-up** |  |  |
| **Agnieszka Janion-Sadowska et al.** | * | * | * | * | * | - | - | * | - | **6** |  |
| **Chun‑Li Wang et al.** | * | * | - | * | - | - | * | * | - | **5** |  |
| **Daniele Pastori et al.** | * | * | * | * | * | - | - | * | * | **7** |  |
| **Eitaro Kodani et al.** | * | * | * | * | * | - | - | * | - | **6** |  |
| **Jiesuck Park et al.** | * | * | - | * | * | * | * | * | - | **7** |  |
| **Tuomas Kiviniemi et al.** | * | * | * | * | * | - | * | - | * | **7** |  |
| **Varun Iyengar, et al.** | * | * | * | * | * | - | * | - | * | **7** |  |
| **Wenlin Xu et al** | * | * | * | * | * | - | * | - | - | **6** |  |
| **Xiaochun Zhang et al.** | * | * | * | * | * | - | * | - | * | **7** |  |
| **Yoav Michowitz et al.** | * | * | - | * | * | - | - | * | - | **5** |  |
| **Yung-Hsin Yeh et al.** | * | * | * | * | * | - | * | - | - | **6** |  |
| **Yurong Xiong et al.** | * | * | - | * | * | - | * | - | - | **5** |  |
| *****indicates criterion met; - indicates significant of criterion not met | | | | | | | | | | |  |

**Supplementary Table S2 Egger test P value**

|  |  |  |  |  |  |  |  |  |
| --- | --- | --- | --- | --- | --- | --- | --- | --- |
| **Outcome** | **Ischemic stroke/ systemic embolism** | **Bleeding event** | **Major bleeding** | **Minor bleeding** | **gastrointestinal hemorrhage** | **Intracranial hemorrhage** | **Clinically relevant non-major bleeding** | **Mortality** |
| **P value** | **0.0891** | **0.0049** | **0.8912** | **0.0024** | **0.5394** | **0.9933** | **0.0997** | **0.2872** |
| p < 0.05 was showed publication bias | | | | | | | | |
